# Fragility index of positive phase II and III randomised clinical trials of treatments for hepatocellular carcinoma (2002–2022)

**DOI:** 10.1016/j.jhepr.2023.100755

**Published:** 2023-04-07

**Authors:** Sabrina Sidali, Nanthara Sritharan, Claudia Campani, Jules Gregory, François Durand, Nathalie Ganne-Carrié, Maxime Ronot, Vincent Lévy, Jean-Charles Nault

**Affiliations:** 1 Université de Paris, Service d’Hépatologie, DMU DIGEST, Hôpital Beaujon, APHP Nord, Clichy, France; 2 Centre de Recherche des Cordeliers, Sorbonne Université, Inserm, Université de Paris, Team ‘Functional Genomics of Solid Tumors’, Equipe Labellisée Ligue Nationale Contre le Cancer, Labex OncoImmunology, Paris, France; 3 Department of Clinical Research, Paris Seine Saint Denis Hospital, Sorbonne Paris University, APHP, Bobigny, France; 4 Department of Radiology, FHU MOSAIC, Hôpital Beaujon APHP Nord, Clichy, France; 5 Université de Paris, INSERM, UMR1153, Epidemiology and Biostatistics Sorbonne Paris Cité Center (CRESS), METHODS Team, Paris, France; 6 Liver Unit, Hôpital Avicenne, Hôpitaux Universitaires Paris-Seine-Saint-Denis, Assistance-Publique Hôpitaux de Paris, Bobigny, France; 7 Unité de Formation et de Recherche Santé Médecine et Biologie Humaine, Université Sorbonne Paris Nord, Bobigny, France; 8 Université de Paris, INSERM U1149 ‘Centre de Recherche sur L'inflammation’, CRI, Paris, France; 9 ECSTRRA Team, CRESS UMR 1153, Hôpital Saint-Louis, APHP, Paris, France

**Keywords:** Fragility index, Fragility quotient, *p* value, Hepatocellular carcinoma, Randomised controlled clinical trial

## Abstract

**Background & Aims:**

The fragility index (FI), i.e., theminimum number of best survivors reassigned to the control group required to revert the statistically significant result of a clinical trial to non-significant, is a metric to evaluate the robustness of randomized controlled trials (RCTs). We aimed to assess the FI in the field of HCC.

**Methods:**

This is a retrospective analysis of phase 2 and 3 RCTs for the treatment of HCC published between 2002 and 2022. We included two-arm studies with 1:1 randomization and significant positive results for a primary time-to-event endpoint for the FI calculation, which involves the iterative addition of a best survivor from the experimental group to the control group, until positive significance (*p* <0,05, Log-rank test) is lost.

**Results:**

We identified 51 phase 2 and 3 positive RCTs, of which 29 (57%) were eligible for fragility index calculation. After reconstruction of the Kaplan-Meier curves, 25/29 studies remained significant, among which the analysis was performed. The median (interquartile range (IQR)) FI was 5 (2-10) and Fragility Quotient (FQ) was 3% (1%-6%). Ten trials (40%) had a FI of 2 or less. FI was positively correlated to the blind assessment of the primary endpoint (median FI 9 with blind assessment versus 2 without, *p* = 0.01), the number of reported events in the control arm (RS = 0.45, *p* = 0.02) and to impact factor (RS = 0.58, *p* = 0.003).

**Conclusions:**

Several phases 2 and 3 RCTs in HCC have a low fragility index, underlying the limited robustness on the conclusion of their superiority over control treatments. The fragility index might provide an additional tool to assess the robustness of clinical trial data in HCC.

**Impact and implications:**

The fragility index is a method to assess robustness of a clinical trial and is defined the minimum number of best survivors reassigned to the control group required to revert the statistically significant result of a clinical trial to non-significant. Among 25 randomised controlled trials in HCC, the median fragility index was 5, and 10 trials among 25 (40%) had a fragility index of 2 or less, indicating an important fragility.

## Highlights

• The fragility index (FI; a method to assess robustness of a trial) is the minimum number of best survivors reassigned to the control group required to revert the statistically significant result of a clinical trial to non-significant.
• Among 25 randomised controlled trials in HCC, the median FI was 5, and 10 trials among 25 (40%) had an FI of 2 or less, indicating an important fragility.
• FI was correlated with the blind assessment of the primary endpoint, the number of reported events in the control arm, and the impact factor.

## Graphical abstract

**Figure:**
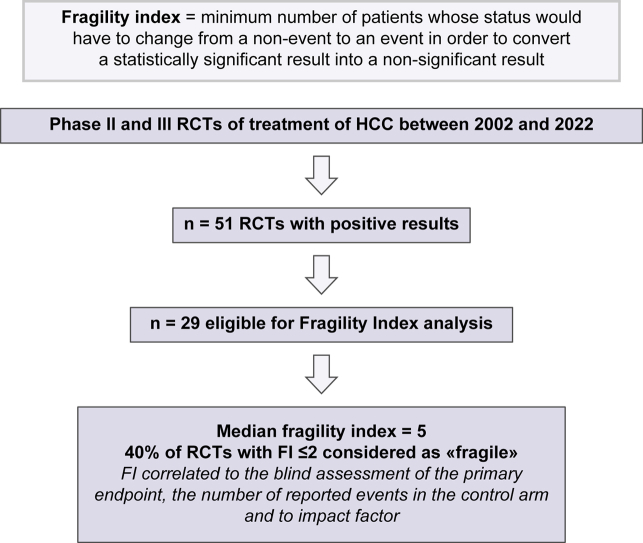

## Introduction

HCC is the third most common cause of cancer-related death and occurs mainly in chronic liver disease at the cirrhosis stage.^1^ The Barcelona Clinic Liver Cancer classification is the most commonly used staging system for HCC in Western countries, linking tumour burden, liver function, and performance status with prognosis and therapeutic management.^2^ In 2022, the Barcelona Clinic Liver Cancer group updated its treatment algorithm to reflect recent advances, especially regarding systemic treatment strategies.^2^ All the treatments of HCC – namely, radiofrequency ablation, transhepatic chemoembolisation, anti-angiogenic tyrosine kinase inhibitors, and immune checkpoint inhibitors, such as atezolizumab (programmed death-ligand 1 [PDL-1] inhibitor) + bevacizumab (antivascular endothelial growth factor) or durvalumab (anti-PDL1 inhibitor) + tremelimumab (cytotoxic T-lymphocyte-associated protein 4 [CTLA4] inhibitor) combinations – were validated in randomised controlled trials (RCTs).

RCTs are designed to assess a specific intervention’s safety and efficacy, and are considered to produce highly reliable evidence if appropriate methodologies are used. Although clinicians often rely on provided *p* values to interpret results and establish significance in RCT results, this practice remains discussed.^3^ In addition to the *p* value, the unit fragility index (FI) offers an easy tool to evaluate the numerical stability of a contrasted difference between two proportions.^4^ Indeed, outcomes that meet the arbitrary threshold of a *p* value less than 0.05 might not be clinically relevant and be based on a low number of events in the experimental arm to reach the significance. The FI was defined as the minimum number of patients whose status would have to change from a non-event to an event required to turn a statistically significant result into a non-significant result.^5^ Bomze *et al.*^6^ introduced a simple and intuitive FI for survival analysis as the minimum number of best survivors reassigned from the experimental group to the control group.^6^ Consequently, the FI has been recommended as an additional statistical method to present and interpret the results of RCTs.

Therefore, our study aimed to assess the FI of positive phase II and III RCTs in the treatment of HCC in the past two decades and identify the characteristics of RCT associated with FI.

## Materials and methods

### Study design and selection of RCTs

To identify positive RCTs relevant to this study, we searched through MEDLINE on PubMed, the Cochrane Library, and the Clinical Trials database using the following terms: ‘hepatocellular carcinoma’ and ‘HCC’, as free text word and/or combined with ‘trial’, ‘prospective’, ‘phase II’, ‘phase 2’, ‘phase III’, ‘phase 3’, ‘randomized’, ‘randomised’, ‘controlled’.

We screened for prospective phase II and III RCTs published between 1 January 2002 and 30 June 2022 with a statistically significant result based on time-to-event data (primary endpoint). We excluded non-inferiority RCTs, RCTs with three arms, RCTs that reported statistically non-significant primary outcomes (*p* ≥0.05), and RCTs without a clear definition of the primary endpoint and their related results. Three reviewers (SS, JCN, and CC) independently screened all identified abstracts and performed data extraction.

### Data extraction

The following characteristics of each study, including RCT phase (II, II/III, or III), were collected: year of publication, journal of publication and impact factor, sample size, number of enrolling centres, disease stages, treatment arms, type of endpoints, outcomes of interest, and response assessment. Studies were stratified according to quality using a modified version of the Jadad score and the Delphi list consisting of five and nine items, respectively.^7^^,^^8^ Studies were defined as high quality with a Jadad score ≥6 and a Delphi score ≥5.

Individual survival data from studies were extracted from the Kaplan–Meier curves published using the Digitizer software application (https://automeris.io/WebPlotDigitizer/).^9^^,^^10^ The reconstructed curves were then compared with the published data to confirm the accuracy of the reconstructed data.

### Statistical analysis and calculation of the FI

We described continuous data (median [IQR]) and categorical data (frequency and percentage). Comparisons of continuous and categorical variables were performed using the Mann–Whitney test, and the Chi-square or Fisher exact test, respectively.

The FI for survival curves was calculated by iterative reassigning the best survivors from the experimental group to the control group until positive significance (defined as *p* <0.05) was lost. The best survivor is defined as the patients with the longest follow-up time, regardless of having an event or being censored.^6^ Values of *p* were assessed using a two-tailed log-rank test. A smaller FI indicates a less robust study result. Some significant studies in the publications that turned out to be non-significant after the reconstruction of the Kaplan–Meier curves were excluded from the main analysis.

To overcome the effect of sample size in interpreting the FI, we calculated the fragility quotient (FQ), which is the FI divided by the sample size.^11^^,^^12^ This would allow us to see what proportion of patients (best survivors) needs to be moved to make the results meaningless or meaningful (the percentage of patients required to be removed to lose the significance). A smaller FQ also indicates a less robust study result.

To evaluate associations between the FI and FQ, and trial characteristics, we used the Spearman rank order correlation coefficient (R_S_) for continuous variables. The Kruskal–Wallis test was used for parameters with more than two modalities, and the Wilcoxon–Mann–Whitney test was used for those with two modalities.

Values of *p* <0.05 were considered significant. Statistical analyses were performed using GraphPad Prism 7.0 (La Jolla, CA, USA) and R Project for Statistical Computing, version 3.5.2 software (The R Foundation for Statistical Computing, Vienna, Austria; http://www.r-project.org/).

## Results

**Fig. 1:**
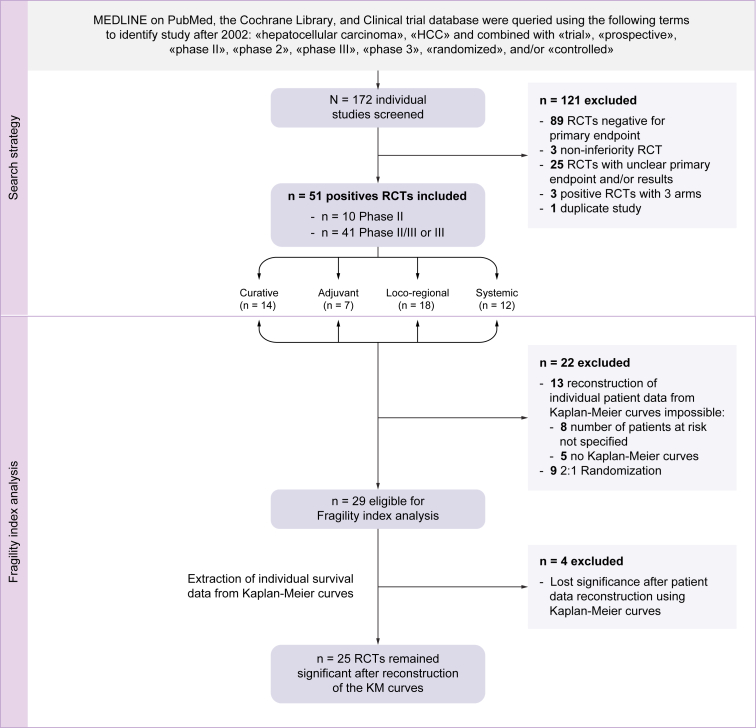
Flow chart of the study and description of the search strategy. We described the search strategy of the RCTs on HCC between 2002 and 2022 that could be included in the fragility index analysis. HCC, hepatocellular carcinoma; KM, Kaplan–Meier; RCT, randomised controlled trial.

A total of 172 phase II and III RCTs published between 1 January 2002 and 30 June 2022 were screened. After the exclusion of 121 studies, 51/172 (29%) were positive with a statistically significant result for a primary time-to-event endpoint and were included in our study (Fig. 1).

### General characteristics of positive phase II and III prospective RCTs

The characteristics of the 51 positive phase II and III prospective RCTs included are summarised in Table 1, Table 2. We identified 37 academic-driven studies and 14 industry-driven studies. Most RCTs were performed in patients with an early or intermediate stage of HCC (n = 41) and in Eastern populations (n = 37). The median Jadad and Delphi scores were 8 (IQR 7–8) and 6 (IQR 5–6), respectively. Forty-three (84%) studies were defined as high-quality studies with a Jadad score of ≥6 and a Delphi list score of ≥5. The median impact factor was 17.96 (IQR 7.11–41.32), and 35/51 RCTs (69%) had an impact factor of >10. Among the 51 positive RCTs, 9 were excluded because of a 2:1 allocation ratio and 13 because of the impossibility of reconstructing individual patient data from published Kaplan–Meier survival curves (eight with number of patients at risk not specified and five without Kaplan–Meier curves) (Fig. 1). Finally, 29 RCTs were eligible for FI calculation (Table 1, Table 2).

**Table 1:** **Description of positive phase II and III prospective RCTs in the treatment of hepatocellular carcinoma between 2002 and 2022**.

	Positive phase II and III prospective RCTs included in the study (N = 51)	RCTs eligible for fragility index calculation (n = 29)	Significant RCTs after reconstruction of KM curves (n = 25∗)
Treatment			
Curative intent	14 (27)	10 (34)	8 (32)
Adjuvant	7 (14)	4 (14)	4 (16)
Non-curative intent locoregional treatment	18 (35)	11 (38)	10 (40)
Systemic	12 (24)	4 (14)	3 (12)
Year of the end of inclusion	2013 (2008–2017)	2014 (2010–2018)	2014 (2010–2017)
Year of publication	2017 (2013–2020)	2018 (2014–2021)	2018 (2014–2021)
Academic study	37 (72%)	24 (86%)	21 (88%)
Impact factor	17.96 (7.11–41.32)	21 (11–34)	21 (11–27)
RCT			
Phase II	10 (20)	6 (21)	5 (20)
Phase II/III	3 (6)	1 (3)	0 (0)
Phase III	38 (74)	22 (76)	20 (80)
Design			
Unicentric	27 (53)	15 (54)	10 (42)
Multicentric	24 (47)	13 (46)	14 (58)
Sample size	173 (88–271)	189 (80–262)	173 (80–250)
OS endpoint	24 (47)	14 (48)	12 (48)
Fragility index	—	—	5 (2–10)
Fragility quotient (%)	—	—	3 (1–6)
Delphi list	6 (5–6)	6 (6–6)	6 (6–6)
Jadad score	8 (7–8)	8 (7–8)	8 (7–8)

Data are presented as counts N (%) or median (IQR).

KM, Kaplan-Meier; OS, overall survival; RCT, randomised controlled trial.

∗ After reconstruction of the KM curves, 25/29 studies remained significant.

**Table 2:** **Characteristics of all the positive phase II and III prospective RCTs in the treatment of HCC between 2022 and 2022 (N** = **51)**.

Trial/first author (year)	Country	Characteristics of the trial	Arms and number of patients per arm	Primary endpoint	Secondary endpoint	Results on primary endpoints	Calculated *p* value∗	Fragility index†	Quality assessment: Delphi list	Quality assessment: Jadad
**Curative treatment**										
Liu *et al.* (2016) (1)	China	HCC within Milan criteria	RFA + TACE (n = 100) Resection (n = 100)	OS	RFS	OS rate at 5 yr 61.9 *vs*. 45.7%, *p* = 0.007	0.008	5	6	8
Wang *et al.* (2015) (2)	China		Percutaneous cryoablation (n = 180) RFA (n = 180)	LTP	Safety, OS, TFS	LTP at 3 yr 7 *vs*. 11%, *p* = 0.043	NS (0.06)	-3	6	7
Morimoto *et al.* (2010) (3)	Japan		RFA + TACE (n = 19) RFA (n = 18)	LTP	Safety, OS, Recurrence rate	9 *vs*. 39%, *p* = 0.012	NA	NA	9	8
Brunello *et al.* (2008) (4)	Italy		RFA (n = 70) Ethanol injection (n = 69)	CR at 1 yr	Survival, early CR, safety	65.7 *vs*. 36.2%, *p* = 0.0005	NA	NA	6	7
Chen *et al.* (2014) (5)	China		RFA (n = 68) RFA-I125 (n = 68)	Recurrence at 5 yr	OS	39.8 *vs*. 57.4%, HR 0.508 (95% CI 0.317–0.815); *p* = 0.004	0.004	6	9	8
Huang *et al.* (2010) (6)	China		RFA (n = 115) Resection (n = 115)	OS	RFS	OS rate at 5 yr 54.8 *vs*. 75.6%, *p* = 0.001	0.0009	14	6	7
Mazzafero *et al.* (2020) (7)	Italy		LT after downstaging (n = 23) Other treatment after downstaging (n = 22)	5-yr survival	Cost–benefit analysis	76.8 *vs*. 18.3%, HR 0.20 (95% CI 0.07–0.57); *p* = 0.003	0.02	2	6	8
Peng *et al.* (2012) (8)	China		TACE after RF (n = 60) RFA alone (n = 70)	OS	RFS	OS rate at 5 yr 46 *vs*. 36%, *p* = 0.037	NA	NA	5	8
Peng *et al.* (2013) (9)	China		TACE after RF (n = 94) RFA alone (n = 95)	OS	RFS, adverse effects	OS rate at 4 yr 61.8 *vs*. 59.5%, HR 0.52 (95% CI 0.335–0.822); *p* = 0.001	NS (0.06)	-1	5	8
Shiina *et al.* (2005) (10)	Japan		RFA (n = 118) Ethanol injection (n = 114)	4-yr OS	Recurrence, LTP	74 *vs*. 57%, *p* = 0.01	0.01	5	5	8
Wei *et al.* (2018) (11)	China	Unifocal HCC ≥5 cm with vascular invasion	Resection + TACE (n = 125) Resection (n = 125)	DFS	OS, safety	17.5 *vs*. 9.3 months, *p* = 0.02	0.02	2	4	6
Yin *et al.* (2014) (12)	China	Resectable multiple HCC beyond Milan criteria	Partial hepatectomy (n = 88) TACE (n = 85)	OS	—	mOS 41 *vs*. 14 months, *p* <0.001	0.2e-05	11	6	7
Zhai *et al.* (2013) (13)	China	Small HCC	THM + resection (n = 180) TACE + resection (n = 184)	RR at 1 yr	Safety	46.9 *vs*. 34.5 months, *p* = 0.048	0.003	2	5	7
Zhong *et al.* (2009) (14)	China	HCC stage IIIA	TACE + resection (n = 57) Resection alone (n = 58)	OS	RR, RFS, safety	mOS 23 *vs*. 14 months, *p* = 0.048	NA	NA	5	6

**Adjuvant treatment**										
Li *et al.* (2020) (15)	China	HCC with microvascular invasion	TAIC with FOLFOX after resection (n = 64) Resection alone (n = 64)	DFS	OS, safety	DFS at 1 yr 61.8 *vs*. 48.1%, *p* = 0.023	0.002	2	6	8
Wang *et al.* (2018) (16)	China	HBV-related HCC with an intermediate or high risk of recurrence	Adjuvant TACE after resection (n = 140) Resection alone (n = 140)	RFS	OS, safety	56 *vs* 42.1%, *p* = 0.01	0.01	4	6	8
Kuang *et al.* (2004) (17)	China	Phase II	AFFTV after resection (n = 19) Placebo (n = 22)	RFS	OS	10.3 *vs*. 6.6 months, *p* = 0.003	NA	NA	5	8
Lee *et al.* (2015) (18)	South Korea	Curative treatment (RFA, ethanol injection, and resection)	Adjuvant immunotherapy with autologous CIK cells (n = 114) No adjuvant treatment (n = 114)	RFS	OS, safety	Immunotherapy > no adjuvant treatment, *p* = 0.08, not reached mOS	0.02	2	6	8
Li *et al.* (2020) (19)	China	Phase II, HCC CD147+	Adjuvant ^131^I-metuximab after resection (n = 78) No adjuvant treatment (n = 78)	5-yr RFS	OS, safety	43.4 *vs*. 21.7%, HR 0.49 (95% CI 0.34–0.72); *p* = 0.0031	2.1e0.5	10	6	8
Chen *et al.* (2013) (20)	China		Iodine-125 after resection (n = 34) Resection alone (n = 34)	TTR	OS	60 *vs*. 36.7 months, *p* = 0.008	NA	NA	5	6
Xu *et al.* (2015) (21)	China		CIK cells after curative resection (n = 100) Resection alone (n = 100)	TTR	DFS, adverse events	13.6 *vs*. 7.8 months, *p* = 0.01	NA	NA	5	6

**Locoregional treatment**										
He *et al.* (2019) (22)	China	HCC with portal invasion	Sorafenib + hepatic arterial infusion of oxaliplatin/5FU/leucovirin (n = 125) Sorafenib alone (n = 122)	OS	PFS, ORR, safety	13.4 *vs*. 7.1 months, HR 0.35 (95% CI 0.26–0.48); *p* = 0.001	1.9e-08	16	6	8
TACTICS Kudo *et al.* (2020) (23)	Japon		TACE + sorafenib (n = 80) TACE alone (n = 76)	PFS	Safety	25.2 *vs*. 13.5 months, *p* = 0.006	0.04	1	6	8
Mohnike *et al.* (2018) (24)	Germany	Phase II	Radioablation by HDRiBT (n = 37) cTACE (n = 40)	TTNTP	Survival, TTP	67.5 *vs*. 27.4%, *p* = 0.019	NS (0.06)	-1	9	8
Ding *et al.* (2021) (25)	China		TACE + lenvatinib (n=32) TACE + sorafenib (n=32)	TTP	OS, ORR, safety	mTTP 4.7 *vs*. 3.1 months; HR 0.55 (95% CI 0.32–0.95); *p* = 0.029	0.01	1	6	8
DOSISPHERE-01 Garin *et al.* (2020) (26)	France	Phase II	SIRT with personalised dosimetry (n = 28) SIRT with standard dosimetry (n = 28)	ORR	OS, PFS, safety, dose response evaluation	78 *vs*. 36%, *p* = 0.0074	NA	NA	6	8
Ikeda *et al.* (2016) (27)	Japan	Phase II	Sorafenib + HAIC with cisp (n = 66) Sorafenib (n = 42)	OS	PFS, RR	10.6 *vs*. 8.7 months, HR 0.60 (95% CI 0.38–0.96); *p* = 0.031	NA	NA	6	8
Kubota *et al.* (2018) (28)	Japan		TACE with mirip (n = 99) TACE with epirub (n = 99)	TTP	RR, safety	mTTP 5.9 *vs*. 7.6 months, *p* = 0.021	NA	NA	6	5
Lo *et al.* (2002) (29)	China	Unresectable HCC	TACE (n = 40) Symptomatic treatment (n = 40)	OS	Tumoral response, liver function, safety	OS at 1 yr 57 *vs*. 32%, *p* = 0.002	0.002	2	5	6
Mabed *et al.* (2009) (30)	Egypt		TACE with lipiodol, doxo, and cisp (n = 50) Intravenous doxo (n = 50)	Response rate	TTP, OS, toxicity	Partial RR 32 *vs*. 10%, *p* = 0.007	NA	NA	4	4
Salem *et al.* (2016) (31)	USA	Phase II	^90^Y Radioembolisation (n = 24) TACE (n = 21)	TTP	Safety, RR, OS	*>26 vs*. 6.8 months, *p* = 0.0012	0.0002	6	6	5
Yamashita *et al.* (2011) (32)	Japan		IFN + HAI of 5FU/cisp (n = 57) IFN + HAI of 5FU alone (n = 57)	Response rate	OS, PFS, adverse effects	45.6 *vs*. 24.6%, *p* = 0.030	NA	NA	4	5
Yoon *et al.* (2018) (33)	South Korea	HCC with macrovascular invasion	TACE + EBR (n = 45) Sorafenib (n = 45)	12-wk PFS	OS, PFS, RR, TTP, time to treatment crossover	86.7 *vs*. 34.3%, *p* = 0.001	5.4e-10	13	7	8
Yang *et al.* (2014) (34)	China	HCC with portal vein thrombosis	TACE + endovascular implantation of an iodine-125 seed strand (n = 43) TACE alone (n = 42)	OS	Tumoural response, post-procedure complications, safety	OS at 180 days 58.9 *vs*. 30.7%, *p* <0.0001	NA	NA	6	7
Li *et al.* (2021) (35)	China		FOLFOX-HAIC (n = 159) TACE (n = 156)	OS	Response, PFS, safety	mOS 23.1 *vs*. 16.1 months, HR 0.58 (95% CI 0.45–0.75); *p* <0.001	2.7e-05	10	5	8
Dhont *et al.* (2022) (36)	Belgium	Phase II	^90^Y Radioembolisation (n = 38) DEB-TACE (n = 34)	TTP	OS, safety	mTTP 17.1 *vs*. 9.5 months, HR 0.36 (95% CI 0.18–0.70); *p* = 0.002	0.003	2	5	5
FOHAIC-1 Liy *et al.* (2022) (37)	China		Arterial chemotherapy of oxaliplatin 5FU (n = 130) Sorafenib (n = 132)	OS	Tumour downstaging, response	mOS 13.9 *vs*. 8.2 months, HR 0.408 (95% CI 0.301–0.552); *p* <0.001	<0.0001	12	6	8
Zheng *et al.* (2022) (38)	China	Phase II, HCC with major portal vein tumour thrombosis	Sorafenib + HAIC (n = 32) Sorafenib (n = 32)	OS	ORR, PFS, safety	mOS 16.3 vs. 6.5 months, HR 0.28 (95% CI 0.150.53); *p* <0.01	<0.001	6	6	8
JIVROSG-1302 Ikeda *et al.* (2022) (39)	Japan		DEB-TACE (n = 99) cTACE (n = 101)	CRR at 3 months	CRR at 1 month, incidence of adverse events	75.3 *vs*. 27.6%, *p* <0.001	NA	NA	5	8

**Systemic treatment**										
SHARP (2008) (40)	International	Western population	Sorafenib (n = 299) Placebo (n = 303)	OS, TTSP	TTP, DCR, safety	10.7 *vs*. 7.9 months, HR 0.69 (95% CI 0.55–0.87); *p* <0.001	0.002	8	10	9
Asia-Pacific (2009) (41)	Taiwan	Eastern population	Sorafenib (n = 150) Placebo (n = 76)	None predefined		6.5 *vs*. 4.2 months, HR 0.68 (95% CI 0.50–0.93); *p* = 0.014	NA	NA	9	9
IMBRAVE-150 (2020) (42,43)	International	No	Atezolizumab/bevacizumab (n = 336) Sorafenib (n = 165)	OS/PFS	ORR, QoL, response duration	19.2 *vs*. 13.4 months, HR 0.66 (95% CI 0.52–0.85); *p* <0.001	NA	NA	7	8
HIMALAYA (2022) (44)	International	No	Durvalumab/tremelimumab (n = 393) Sorafenib (n = 389)	OS	Non-inferiority OS for durvalumab *vs*. sorafenib	16.4 *vs*. 13.8 months, HR 0.78 (95% CI 0.65–0.92); *p* = 0.0035	0.004	8	6	5
RESORCE (2017) (45)	International	Patients tolerant to sorafenib	Regorafenib (n = 379) Placebo (n = 194)	OS	PFS, TTP, ORR, DCR	10.6 *vs*. 7.8 months, HR 0.63 (95% CI 0.50–0.79); *p* <0.001	NA	NA	9	10
CELESTIAL (2018) (46)	International	No	Cabozantinib (n = 470) Placebo (n = 237)	OS	PFS, ORR	10.2 *vs*. 8.0 months, HR 0.76 (95% CI 0.63–0.92); *p* = 0.005	NA	NA	9	10
REACH-2 (2019) (47)	International	Patients with serum AFP >400 ng/ml	Ramucirumab (n = 197) Placebo (n = 95)	OS	PFS, TTP, ORR, safety	8.5 *vs*. 7.3 months, HR 0.71 (95% CI 0.53–0.94); *p* = 0.0199	NA	NA	9	10
ALHEP (2021) (48)	China	RCT in China, second-line or later therapy	Apatinib (n = 267) Placebo (n = 133)	OS	Safety	8.7 *vs*. 6.8 months, HR 0.785 (95% CI 0.617–0.998); *p* = 0.048	NA	NA	5	8
Qin *et al.* (2021) (49)	China	Phase II/III	Donafenib (n = 328) Placebo (n = 331)	OS	PFS, TTP, ORR, DCR, safety	12.1 *vs*. 10.3 months, HR 0.831 (95% CI 0.699–0.988); p = 0.0245	NS (0.05)	-1	6	4
Ryoo *et al.* (2021) (50)	South Korea, China, Taiwan	Phase Ib/II, Eastern population, HCC with MET overexpression	Tepotinib (n = 38) Placebo (n= 37)	TTP	PFS, OS, safety, DCR, ORR,	2.9 *v*s. 1.4 months, HR 0.42 (95% CI 0.26–0.70); *p* = 0.0043	0.003	2	6	8
ORIENT-32 Ren *et al.* (2021) (51)	China	Phase II/III	Sintilimab/bevacizumab (n = 380) Sorafenib (n = 191)	OS	PFS, ORR, DCR, TTP, time to deterioration of health status, immunogenicity of sintilimab	mOS not reached *vs*. 10.4 months for sorafenib; PFS 4.6 *vs*. 2.8 months	NA	NA	5	8
Santoro *et al.* (2013) (52)	Italy	Second-line treatment, after progression and/or poor tolerance of first line for HCC with MET overexpression	Tivantinib (n = 71) Placebo (n = 36)	TTP	OS, PFS, safety, DCR, ORR	1.6 *vs*. 1.4 months, HR 0.64 (95% CI 0.19–0.97); *p* = 0.04	NA	NA	9	10

5FU, fluorouracil; AFFTV, autologous formalin-fixed tumour vaccine; AFP, alpha fetoprotein; CIK, cytokine-induced killer; cisp, cisplatin; CR, complete response; CRR, complete response rate; DCR, disease control rate; DEB-TACE, drug-eluting bead TACE; DFS, disease-free survival; DOR, duration of response; doxo, doxorubicin; EBR, external beam radiotherapy; epirub, epirubicin; FOLFOX, 5-fluorouracil and oxaliplatin; HAI, hepatic arterial infusion; HAIC, hepatic arterial infusion chemotherapy; HDRiBT, high-dose-rate interstitial brachytherapy; HR, hazard ratio; IFN, interferon; LT, liver transplantation; LTP, local tumour progression; mirip, miriplatin; mOS, median OS; mTTP, median TTP; NA, not available; ORR, objective response rate; OS, overall survival; PFS, progression-free survival; QoL, quality of life; RCT, randomised controlled trial; RFA, radiofrequency ablation; RFA-I125, RFA and percutaneous iodine-125; RR, recidive rate; SIRT, selective internal radiation therapy; TACE, transhepatic chemoembolisation; cTACE, conventional TACE; TAIC, transarterial infusion chemotherapy; TFS, tumour-free survival; THM, traditional herbal medicine; TTNTP, time to not treatable progression; TTP, time to tumour progression; TTR, time to response; TTSP, time to symptomatic progression.

∗ Log-rank test.

† Fragility Index analysis was possible for 29 RCTs. After the reconstruction of the Kaplan–Meier curves, 25/29 studies remained significant and were included in the main statistical analysis. See Supplementary information for the references of all the trials.

### FI analysis

Among the 29 studies with a 1:1 allocation ratio eligible for FI calculation (see Table 1 for the characteristics of these studies), 13 were multicentric (46%), mostly performed in patients with an early or intermediate stage of HCC (88%) and in Eastern populations (79%). The median Jadad and Delphi scores were 8 (IQR 7–8) and 6 (IQR 6–6), respectively.

After the reconstruction of the Kaplan–Meier curves, 25/29 studies remained significant, and four studies had a non-significant *p* value. Among these four studies, the *p* value was evaluated using Cox proportional hazards regression models and not using the log-rank test for three studies,[13], [14], [15] and for the last study,^16^ the *p* value was assessed using a stratified log-rank test with random assignment stratifications factors.

**Fig. 2:**
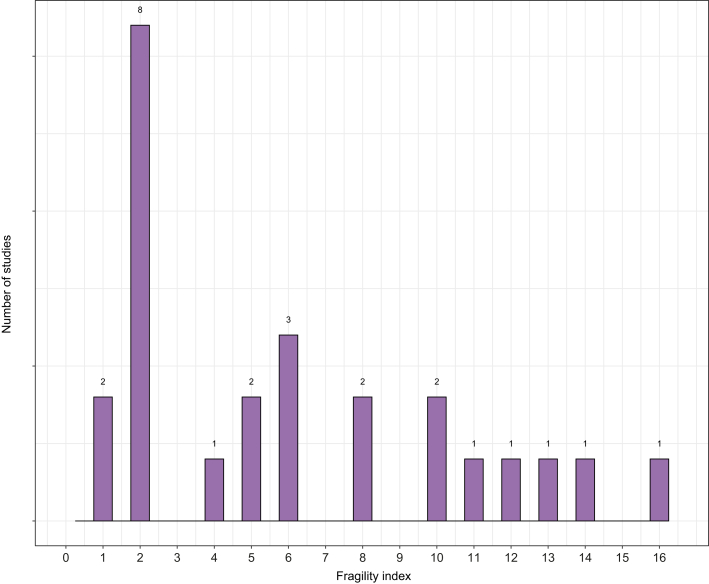
Distribution of fragility index across studies (N = 25). We figured the distribution of the fragility index of the 25 randomised controlled trials finally included in the analysis.

Among the 25 studies with a remaining significant *p* value after the reconstruction of the Kaplan–Meier curves (see Table 1 for the characteristics of these studies), the median FI was 5 (IQR 2–10), and the median FQ was 3% (IQR 1–6%). Ten studies had an FI of ≤2. The distribution of the FI of the remaining 25 studies is represented in Fig. 2. We performed subgroup analysis according to the types of treatment received: curative intent treatment (n = 8; median FI 5 [IQR 2–7.2]), adjuvant treatment (n = 4; median FI 3 [IQR 2–5.5]), locoregional treatments in a non-curative intent (n = 10; median FI 5 [IQR 2–11.5]), and systemic treatments in advanced stages (n = 3; median FI 8 [IQR 5–8]) (*p* = 0.9, Kruskal–Wallis non-parametric test). To note, among the nine positive RCTs not initially included in the FI calculation because of the inability to perform correlation with trial features because of a 2:1 randomisation ratio, seven remained significant after reconstruction of Kaplan–Meier curves, and for these studies, the median FI and median FQ were 4 (IQR 2.5–14.5) and 1% (IQR 0.6–2%), respectively.

Among the 25 studies included in the FI analysis, FI was associated with a blind assessment of the primary endpoint (median FI 9 [IQR 8–12] with blind assessment *vs.* 2 [IQR 2–6] without blind assessment; *p* = 0.01). FI was also positively correlated with the number of reported events in the control arm (R_S_ = 0.45, *p* = 0.02) and the impact factor (R_S_ = 0.58, *p* = 0.003) and was negatively correlated with the *p* value (R_S_ = -0.83, *p* <0.0001) (Table 2). There was no significant correlation between the size of the experimental or control group and the FI, and there was no difference in terms of FI between academic and industrial promotion of the study and across the type of treatment assessed (curative, adjuvant, non-curative locoregional, and systemic) (Table 3).

**Table 3:** **Associations between trial features (n** = **25) and FI and FQ**. FI, fragility index; FQ, fragility quotient; R_S_, Spearman correlation.

Variables associated with the FI		
Variables	Correlation R_S_	*p* value
Number of patients in the control arm	0.37	0.07
Number of events in the control arm	0.45	0.02
Number of patients in the experimental arm	0.36	0.08
Number of events in the experimental arm	0.2	0.3
*p* value (log-rank test)	-0.83	<0.0001
Sample size of the study	0.36	0.08
Impact factor	0.58	0.003
Delphi score	0.35	0.09
Jaded score	-0.02	0.9

**Variables**	**Median FI (IQR)**	***p* value**

Blind assessment	9 (8–12.2) with *vs.* 2 (2–6) without	0.01
Academic vs industrial	5 (2–10) academic *vs*. 2 (2–5) industrial	0.5
Curative treatment	5 (2–7.2)	0.9
Adjuvant treatment	3 (2–5.5)	
Non-curative locoregional treatment	6 (2–11.5)	
Systemic treatment	8 (5–8)	

**Variables associated with the FQ**		

**Variables**	**Correlation R**_**S**_	***p* value**

Number of patients in the control arm	-0.42	0.04
Number of events in the control arm	-0.29	0.12
Number of patients in the experimental arm	-0.43	0.03
Number of events in the experimental arm	-0.48	0.02
*p* value (log-rank test)	-0.81	<0.0001
Impact factor	0.38	0.07
Delphi score	0.3	0.1
Jaded score	-0.1	0.6

**Variables**	**Median FQ (IQR) (%)**	***p* value**

Blind assessment	5.2 (2.1–6.3) with *vs*. 2.5 (1.5–4.5) without	0.4
Academic *vs*. industrial	2.8 (1.6–6) academic *vs*. 1 (1–1.8) industrial	0.2
Curative treatment	3.5 (1.8–4.9)	0.3
Adjuvant treatment	1.5 (1.3–2.8)	
Non-curative locoregional treatment	3.9 (2.6–8.7)	
Syste^a^mic treatment	1.3 (1.2–2)	

a Statistical tests use: Chi2 or Fisher exact test for dichotomous variables, Spearman rank order to assess the correlation coefficient (R_s_) and Kruskal-Wallis test and Wilcoxon-Mann-Whitney test for continous variables.

Next, we focused on the correlation between the FQ and the characteristics of clinical trials. The FQ (%) was significantly different between phase II and III studies (median FQ 6.4 [IQR 2.8–9.4] in phase II *vs.* 2.3 [IQR 1.3–4.5] in phase III; *p* = 0.045). In addition, FQ was negatively correlated with the *p* value (R_S_ = -0.81, *p* <0.0001), the number of patients in the experimental arm (R_S_ = -0.43, *p* = 0.03), the number of reported events in the experimental arm (R_S_ = -0.48, *p* = 0.02), and the number of patients in the control arm (R_S_ = -0.42, *p* = 0.04) (Table 3).

## Discussion

The FI is an easy method to quantify the robustness of a trial but should be interpreted with other parameters reported in RCTs such as *p* value, hazard ratio, absolute difference, and power. Moreover, the effect size is often unstable in small trials, and loss to follow-up can decrease confidence in the significance of the effect. The FI is an absolute measure of stability, irrespective of trial size, and we also included in our study the FQ (defined by the absolute FI number divided by the total sample size) to consider the trial sample size.

Our study assessed the FI and FQ of phase II and III RCTs on the treatment of HCC available in the literature between 2002 and 2022. To our knowledge, this is the largest systematic review evaluating the FI and FQ of RCTs to assess the quality of trials in the field of HCC treatment. Among the 51 positive phase II and III prospective RCTs we identified, only 29 were eligible for the calculation of the FI, 4 of which lost significance after patient data reconstruction using Kaplan–Meier curves. The use in the original study of a stratified log-rank test or a Cox proportional hazards model may explain the differences that we found after the reconstruction of Kaplan–Meier curves for these four RCTs. We could also hypothesise that the results of these studies have limited robustness as the significance of the main results vary according to the statistical test performed.

The main findings of our study are as follows: (1) the median FI in positive RCTs in HCC treatments was 5, and the median FQ was 3%; (2) FI was positively correlated with a blind assessment of the primary endpoint, the number of reported events in the control arm, and the impact factor, and was negatively correlated with the *p* value; and (3) FQ was negatively correlated with the *p* value, the number of patients and number of reported events in the experimental arm, and the number of patients in the control arm.

In our study, the median FI was 5, which indicates that at least five best survivors from the experimental arm must be reassigned to the control arm to change the statistically significant result to a non-significant result. As FI is an absolute measure and does not consider the sample size, we calculated the FQ, which is the FI divided by the sample size.^11^^,^^12^ This would allow us to see the proportion of patients (best survivors) that needs to be moved to make the results meaningless or meaningful. A smaller FQ also indicates a less robust study result. The median FQ in our study was 3%; consequently, 3% of the participants should be reassigned to lose significance. Overall, the larger the FI and FQ, the more robust the trial’s results.

Our median FI is slightly higher than the median FI of 2 recently reported by Del Paggio and Tannock^17^ in phase III RCTs of FDA-approved anticancer drugs globally (drugs approved by the FDA between 1 January 2014 and 31 December 2018). Only one study had already assessed the FI in RCT in the HCC field but only included only six RCTs in its analysis, decreasing the applicability of their results.^18^ Moreover, FI has been applied to other RCTs such as oncology, critical care, or heart failure, showing that several RCTs were considered fragile, regardless of the field of research.^11^^,^[19], [20], [21] Several investigators have recommended the routine inclusion of the FI in reporting clinical trial outcomes and developing clinical guidelines.^11^ Although an FI value of 1 indicates extreme fragility, there is no specific cut-off value or lower limit of the FI to classify a study as ‘either fragile’ or ‘robust’. In our study, two RCTs had an FI value of 0–1, indicating extreme fragility, and 10 RCTs had an FI of ≤2, which could be considered as ‘fragile’ RCTs.

FI was also correlated with the impact factor (*p* = 0.003). In a recent study, out of all 2,544 RCTs published between 2014 and 2021 in five high-impact journals (*New England Journal of Medicine*, *The Lancet*, *Journal of the American Medical Association*, *British Medical Journal*, and *Annals of Internal Medicine*), 643 eligible for FI analysis revealed that statistical significance was dependent on a median of 12 (IQR 3–28) events.^22^ In the past decade, statistical significance of RCTs in high-impact journals has become more robust. However, 25% of RCTs are still dependent on three or fewer outcome events.^22^ In addition, the impact factor of journals is not a valid measure of RCT quality, contrary to the Jadad score^7^ and Delphi list,^8^ which were not correlated with the FI in our study. Moreover, FI was higher in RCTs with a blind assessment, suggesting more robust results in these trials. This corroborates evidence in the literature showing that unblind assessment of an endpoint is subject to bias. Moreover, we observed no significant difference in terms of median FI between the types of clinical trials (curative intent treatment, adjuvant treatment, locoregional treatments in a non-curative intent, and systemic treatments in advanced stages). However, the low number of studies included in each subgroup decreases the robustness of this analysis.

Although the FI may improve our understanding of trial results, this method has some limitations, one of which is that the FI can only be calculated in the context of an RCT when outcomes are compared between two groups. Furthermore, the interpretation of the FI can be problematic when the number of participants who drop out for unknown reasons is large. RCTs with small samples and RCTs in which the event of interest is rare tend to be fragile. Another limitation of this study is the inclusion of RCTs characterised by a two-arm parallel design or two-by-two factorial design and with available Kaplan–Meier curves with time-to-event data for FI measurement. Consequently, we did not assess the FI of RCTs with a non-inferiority design and RCTs including more than two arms. This may lead to some uncertainty in generalising our data to all RCTs available in the field of HCC treatments.

However, in our study, we used an adequate statistical methodology for survival data. Indeed, the reconstruction of individual patient data from published Kaplan–Meier curves allowed us to consider not only the events but also the timing of events, which is an essential piece of information to evaluate the effect of treatment on these types of endpoints. A statistical test (log-rank test) adapted to the survival data was also used to evaluate the *p* value and calculate an unbiased FI. Indeed, the original FI proposed by Walsh *et al.*^5^ is based on binary results and the Fisher exact test, which could provide incorrect results for time-to-event data.

In conclusion, our study suggests that several phase II and III RCTs in HCC treatment have a low FI, resulting in uncertainty regarding their robustness and potential clinical benefit. A systematic calculation of the FI could help interpret RCTs and guide their application in daily practice for patients with HCC.

## Financial support

This study received no financial support.

## Authors’ contributions

Contributions to conception and design: SS, MR, VL, JCN. Acquisition of data and/or analysis and interpretation of data: SS, NS, CC, JG, MR, VL, JCN. Drafting and revision of the manuscript content: SS, JCN. Final approval of the version to be published: SS, NS, CC, JG, FD, NG-C, MR, VL, JCN.

## Data availability statement

Not applicable.

## Conflicts of interest

JCN has received research funding from Bayer and Ipsen. SS, NS, CC, JG, and FD have no conflicts of interest. NG-C has received honoraria from Abbie, Bayer, Gilead, Ipsen, Roche, and Shionogi. MR has received educational fees from Canon Medical System, GE Healthcare, Ipsen, Guerbet, and Sirtex. VL has no conflicts of interest.

Please refer to the accompanying ICMJE disclosure forms for further details.
