# Supplementary material for: Fragility index of positive phase II and III randomised clinical trials of treatments for hepatocellular carcinoma (2002–2022)
Source: JHEP Rep. 2023 Apr 7;5(7):100755. doi: 10.1016/j.jhepr.2023.100755 (PMC10326696; doi:10.1016/j.jhepr.2023.100755)
Supplement: Multimedia component 1 [file mmc1.pdf]

Fragility index of positive phase II and III randomized clinical trials of treatments for hepatocellular carcinoma (2002-2022)

Sabrina Sidali, Nanthara Sritharan, Claudia Campani, Jules Gregory, François Durand, Nathalie Ganne-Carrié, Maxime Ronot, Vincent Lévy, Jean-Charles Nault

Supplementary references

1. Liu H, Wang ZG, Fu SY, Li AJ, Pan ZY, Zhou WP, et al. Randomized clinical trial of chemoembolization plus radiofrequency ablation *versus* partial hepatectomy for hepatocellular carcinoma within the Milan criteria. Br J Surg. 18 févr 2016;103(4):348-56.

2. Wang C, Wang H, Yang W, Hu K, Xie H, Hu KQ, et al. Multicenter randomized controlled trial of percutaneous cryoablation versus radiofrequency ablation in hepatocellular carcinoma. Hepatology. 2015;61(5):1579-90.

3. Morimoto M, Numata K, Kondou M, Nozaki A, Morita S, Tanaka K. Midterm outcomes in patients with intermediate-sized hepatocellular carcinoma. Cancer. 2010;116(23):5452-60.

4. Brunello F, Veltri A, Carucci P, Pagano E, Ciccone G, Moretto P, et al. Radiofrequency ablation versus ethanol injection for early hepatocellular carcinoma: A randomized controlled trial. Scand J Gastroenterol. janv 2008;43(6):727-35.

5. Chen K, Chen G, Wang H, Li H, Xiao J, Duan X, et al. Increased survival in hepatocellular carcinoma with iodine-125 implantation plus radiofrequency ablation: A prospective randomized controlled trial. J Hepatol. 1 déc 2014;61(6):1304-11.

6. Huang J, Yan L, Cheng Z, Wu H, Du L, Wang J, et al. A Randomized Trial Comparing Radiofrequency Ablation and Surgical Resection for HCC Conforming to the Milan Criteria: Ann Surg. déc 2010;252(6):903-12.

7. Mazzaferro V, Citterio D, Bhoori S, Bongini M, Miceli R, De Carlis L, et al. Liver transplantation in hepatocellular carcinoma after tumour downstaging (XXL): a randomised, controlled, phase 2b/3 trial. Lancet Oncol. juill 2020;21(7):947-56.

8. Peng ZW, Zhang YJ, Liang HH, Lin XJ, Guo RP, Chen MS. Recurrent hepatocellular carcinoma treated with sequential transcatheter arterial chemoembolization and RF ablation versus RF ablation alone: a prospective randomized trial. Radiology. févr 2012;262(2):689-700.

9. Peng ZW, Zhang YJ, Chen MS, Xu L, Liang HH, Lin XJ, et al. Radiofrequency Ablation With or Without Transcatheter Arterial Chemoembolization in the Treatment of Hepatocellular Carcinoma: A Prospective Randomized Trial. J Clin Oncol. févr 2013;31(4):426-32.

10. Shiina S, Teratani T, Obi S, Sato S, Tateishi R, Fujishima T, et al. A Randomized Controlled Trial of Radiofrequency Ablation With Ethanol Injection for Small Hepatocellular Carcinoma. Gastroenterology. juill 2005;129(1):122-30.

11. Wei W, Jian PE, Li SH, Guo ZX, Zhang YF, Ling YH, et al. Adjuvant transcatheter arterial chemoembolization after curative resection for hepatocellular carcinoma patients with solitary tumor and microvascular invasion: a randomized clinical trial of efficacy and safety. Cancer Commun. 2018;38(1):61.

12. Yin L, Li H, Li AJ, Lau WY, Pan Z ya, Lai ECH, et al. Partial hepatectomy vs. transcatheter arterial chemoembolization for resectable multiple hepatocellular carcinoma beyond Milan criteria: A RCT. J Hepatol. 1 juill 2014;61(1):82-8.

13. Zhai X feng, Chen Z, Li B, Shen F, Fan J, Zhou W ping, et al. Traditional herbal medicine in preventing recurrence after resection of small hepatocellular carcinoma: a multicenter randomized controlled trial. J Integr Med. 1 mars 2013;11(2):90-100.

14. Zhong C, Guo R ping, Li J qing, Shi M, Wei W, Chen M shan, et al. A randomized controlled trial of hepatectomy with adjuvant transcatheter arterial chemoembolization versus hepatectomy alone for Stage III A hepatocellular carcinoma. J Cancer Res Clin Oncol. oct 2009;135(10):1437-45.

15. Li S, Mei J, Wang Q, Guo Z, Lu L, Ling Y, et al. Postoperative Adjuvant Transarterial Infusion Chemotherapy with FOLFOX Could Improve Outcomes of Hepatocellular Carcinoma Patients with Microvascular Invasion: A Preliminary Report of a Phase III, Randomized Controlled Clinical Trial. Ann Surg Oncol. 1 déc 2020;27(13):5183-90.

16. Wang Z, Ren Z, Chen Y, Hu J, Yang G, Yu L, et al. Adjuvant Transarterial Chemoembolization for HBV-Related Hepatocellular Carcinoma After Resection: A Randomized Controlled Study. Clin Cancer Res. 1 mai 2018;24(9):2074-81.

17. Kuang M, Peng BG, Lu MD, Liang LJ, Huang JF, He Q, et al. Phase II Randomized Trial of Autologous Formalin-Fixed Tumor Vaccine for Postsurgical Recurrence of Hepatocellular Carcinoma. Clin Cancer Res. 1 mars 2004;10(5):1574-9.

18. Lee JH, Lee JH, Lim YS, Yeon JE, Song TJ, Yu SJ, et al. Adjuvant Immunotherapy With Autologous Cytokine-Induced Killer Cells for Hepatocellular Carcinoma. Gastroenterology. juin 2015;148(7):1383-1391.e6.

19. Li J, Xing J, Yang Y, Liu J, Wang W, Xia Y, et al. Adjuvant 131I-metuximab for hepatocellular carcinoma after liver resection: a randomised, controlled, multicentre, open-label, phase 2 trial. Lancet Gastroenterol Hepatol. juin 2020;5(6):548-60.

20. Chen K, Xia Y, Wang H, Xiao F, Xiang G, Shen F. Adjuvant Iodine-125 Brachytherapy for Hepatocellular Carcinoma after Complete Hepatectomy: A Randomized Controlled Trial. PLoS ONE. 28 févr 2013;8(2):e57397.

21. Xu L, Wang J, Kim Y, Shuang Z yu, Zhang Y jun, Lao X ming, et al. A randomized controlled trial on patients with or without adjuvant autologous cytokine-induced killer cells after curative resection for hepatocellular carcinoma. Oncoimmunology. 12 oct 2015;5(3):e1083671.

22. He M, Li Q, Zou R, Shen J, Fang W, Tan G, et al. Sorafenib Plus Hepatic Arterial Infusion of Oxaliplatin, Fluorouracil, and Leucovorin vs Sorafenib Alone for Hepatocellular Carcinoma With Portal Vein Invasion: A Randomized Clinical Trial. JAMA Oncol. 1 juill 2019;5(7):953-60.

23. Kudo M, Ueshima K, Ikeda M, Torimura T, Tanabe N, Aikata H, et al. Randomised, multicentre prospective trial of transarterial chemoembolisation (TACE) plus sorafenib as compared with TACE alone in patients with hepatocellular carcinoma: TACTICS trial. Gut. août 2020;69(8):1492-501.

24. Mohnike K, Steffen IG, Seidensticker M, Hass P, Damm R, Peters N, et al. Radioablation by Image-Guided (HDR) Brachytherapy and Transarterial Chemoembolization in Hepatocellular Carcinoma: A Randomized Phase II Trial. Cardiovasc Intervent Radiol. 1 févr 2019;42(2):239-49.

25. Ding X, Sun W, Li W, Shen Y, Guo X, Teng Y, et al. Transarterial chemoembolization plus lenvatinib versus transarterial chemoembolization plus sorafenib as first-line treatment for hepatocellular carcinoma with portal vein tumor thrombus: A prospective randomized study. Cancer. 2021;127(20):3782-93.

26. Garin E, Tselikas L, Guiu B, Chalaye J, Edeline J, de Baere T, et al. Personalised versus standard dosimetry approach of selective internal radiation therapy in patients with locally advanced hepatocellular carcinoma (DOSISPHERE-01): a randomised, multicentre, open-label phase 2 trial. Lancet Gastroenterol Hepatol. janv 2021;6(1):17-29.

27. Ikeda M, Shimizu S, Sato T, Morimoto M, Kojima Y, Inaba Y, et al. Sorafenib plus hepatic arterial infusion chemotherapy with cisplatin versus sorafenib for advanced hepatocellular carcinoma: randomized phase II trial. Ann Oncol. nov 2016;27(11):2090-6.

28. Kubota K, Hidaka H, Nakazawa T, Okuwaki Y, Yamane K, Inoue T, et al. Prospective, randomized, controlled study of the efficacy of transcatheter arterial chemoembolization with miriplatin for hepatocellular carcinoma. Hepatol Res. 2018;48(3):E98-106.

29. Lo CM, Ngan H, Tso WK, Liu CL, Lam CM, Poon RTP, et al. Randomized controlled trial of transarterial lipiodol chemoembolization for unresectable hepatocellular carcinoma. Hepatology. 2002;35(5):1164-71.

30. Mabed M, Esmaeel M, El-Khodary T, Awad M, Amer T. A randomized controlled trial of transcatheter arterial chemoembolization with lipiodol, doxorubicin and cisplatin versus intravenous doxorubicin for patients with unresectable hepatocellular carcinoma. Eur J Cancer Care (Engl). 2009;18(5):492-9.

31. Salem R, Gordon AC, Mouli S, Hickey R, Kallini J, Gabr A, et al. Y90 Radioembolization Significantly Prolongs Time to Progression Compared With Chemoembolization in Patients With Hepatocellular Carcinoma. Gastroenterology. déc 2016;151(6):1155-1163.e2.

32. Yamashita T, Arai K, Sunagozaka H, Ueda T, Terashima T, Yamashita T, et al. Randomized, Phase II Study Comparing Interferon Combined with Hepatic Arterial Infusion of Fluorouracil plus Cisplatin and Fluorouracil Alone in Patients with Advanced Hepatocellular Carcinoma. Oncology. 2011;81(5-6):281-90.

33. Yoon SM, Ryoo BY, Lee SJ, Kim JH, Shin JH, An JH, et al. Efficacy and Safety of Transarterial Chemoembolization Plus External Beam Radiotherapy vs Sorafenib in Hepatocellular Carcinoma With Macroscopic Vascular Invasion. JAMA Oncol. mai 2018;4(5):661-9.

34. Yang M, Fang Z, Yan Z, Luo J, Liu L, Zhang W, et al. Transarterial chemoembolisation (TACE) combined with endovascular implantation of an iodine-125 seed strand for the treatment of hepatocellular carcinoma with portal vein tumour thrombosis versus TACE alone: a two-arm, randomised clinical trial. J Cancer Res Clin Oncol. févr 2014;140(2):211-9.

35. Li QJ, He MK, Chen HW, Fang WQ, Zhou YM, Xu L, et al. Hepatic Arterial Infusion of Oxaliplatin, Fluorouracil, and Leucovorin Versus Transarterial Chemoembolization for Large Hepatocellular Carcinoma: A Randomized Phase III Trial. J Clin Oncol. 14 oct 2021;JCO.21.00608.

36. Dhondt E, Lambert B, Hermie L, Huyck L, Vanlangenhove P, Geerts A, et al. 90Y Radioembolization versus Drug-eluting Bead Chemoembolization for Unresectable Hepatocellular Carcinoma: Results from the TRACE Phase II Randomized Controlled Trial. Radiology. juin 2022;303(3):699-710.

37. Lyu N, Wang X, Li JB, Lai JF, Chen QF, Li SL, et al. Arterial Chemotherapy of Oxaliplatin Plus Fluorouracil Versus Sorafenib in Advanced Hepatocellular Carcinoma: A Biomolecular Exploratory, Randomized, Phase III Trial (FOHAIC-1). J Clin Oncol Off J Am Soc Clin Oncol. 10 févr 2022;40(5):468-80.

38. Zheng K, Zhu X, Fu S, Cao G, Li WQ, Xu L, et al. Sorafenib Plus Hepatic Arterial Infusion Chemotherapy versus Sorafenib for Hepatocellular Carcinoma with Major Portal Vein Tumor Thrombosis: A Randomized Trial. Radiology. mai 2022;303(2):455-64.

39. Ikeda M, Arai Y, Inaba Y, Tanaka T, Sugawara S, Kodama Y, et al. Conventional or Drug-Eluting Beads? Randomized Controlled Study of Chemoembolization for Hepatocellular Carcinoma: JIVROSG-1302. Liver Cancer. 2022;11(5):440-50.

40. Llovet JM, Hilgard P, de Oliveira AC, Forner A, Zeuzem S, Galle PR, et al. Sorafenib in Advanced Hepatocellular Carcinoma. N Engl J Med. 2008;13.

41. Cheng AL, Kang YK, Chen Z, Tsao CJ, Qin S, Kim JS, et al. Efficacy and safety of sorafenib in patients in the Asia-Pacific region with advanced hepatocellular carcinoma: a phase III randomised, double-blind, placebo-controlled trial. Lancet Oncol. janv 2009;10(1):25-34.

42. Cheng AL, Qin S, Ikeda M, Galle PR, Ducreux M, Kim TY, et al. Updated efficacy and safety data from IMbrave150: Atezolizumab plus bevacizumab vs. sorafenib for unresectable hepatocellular carcinoma. J Hepatol. avr 2022;76(4):862-73.

43. Finn RS, Qin S, Ikeda M, Galle PR, Ducreux M, Kim TY, et al. Atezolizumab plus Bevacizumab in Unresectable Hepatocellular Carcinoma. N Engl J Med. 14 mai 2020;382(20):1894-905.

44. Abou-Alfa GK, Chan SL, Kudo M, Lau G, Kelley RK, Furuse J, et al. Phase 3 randomized, open-label, multicenter study of tremelimumab (T) and durvalumab (D) as first-line therapy in patients (pts) with unresectable hepatocellular carcinoma (uHCC): HIMALAYA. J Clin Oncol. févr 2022;40(4_suppl):379-379.

45. Bruix J, Qin S, Merle P, Granito A, Huang YH, Bodoky G, et al. Regorafenib for patients with hepatocellular carcinoma who progressed on sorafenib treatment (RESORCE): a randomised, double-blind, placebo-controlled, phase 3 trial. The Lancet. janv 2017;389(10064):56-66.

46. Abou-Alfa GK, Meyer T, Cheng AL, El-Khoueiry AB, Rimassa L, Ryoo BY, et al. Cabozantinib in Patients with Advanced and Progressing Hepatocellular Carcinoma. N Engl J Med. 5 juill 2018;379(1):54-63.

47. Zhu AX, Park JO, Ryoo BY, Yen CJ, Poon R, Pastorelli D, et al. Ramucirumab versus placebo as second-line treatment in patients with advanced hepatocellular carcinoma following first-line therapy with sorafenib (REACH): a randomised, double-blind, multicentre, phase 3 trial. Lancet Oncol. juill 2015;16(7):859-70.

48. Qin S, Li Q, Gu S, Chen X, Lin L, Wang Z, et al. Apatinib as second-line or later therapy in patients with advanced hepatocellular carcinoma (AHELP): a multicentre, double-blind, randomised, placebo-controlled, phase 3 trial. Lancet Gastroenterol Hepatol. juill 2021;6(7):559-68.

49. Qin S, Bi F, Gu S, Bai Y, Chen Z, Wang Z, et al. Donafenib Versus Sorafenib in First-Line Treatment of Unresectable or Metastatic Hepatocellular Carcinoma: A Randomized, Open-Label, Parallel-Controlled Phase II-III Trial. J Clin Oncol Off J Am Soc Clin Oncol. 20 sept 2021;39(27):3002-11.

50. Ryoo BY, Cheng AL, Ren Z, Kim TY, Pan H, Rau KM, et al. Randomised Phase 1b/2 trial of tepotinib vs sorafenib in Asian patients with advanced hepatocellular carcinoma with MET overexpression. Br J Cancer. 20 juill 2021;125(2):200-8.

51. Ren Z, Xu J, Bai Y, Xu A, Cang S, Du C, et al. Sintilimab plus a bevacizumab biosimilar (IBI305) versus sorafenib in unresectable hepatocellular carcinoma (ORIENT-32): a randomised, open-label, phase 2–3 study. Lancet Oncol. 1 juill 2021;22(7):977-90.

52. Santoro A, Rimassa L, Borbath I, Daniele B, Salvagni S, Laethem JLV, et al. Tivantinib for second-line treatment of advanced hepatocellular carcinoma: a randomised, placebo-controlled phase 2 study. Lancet Oncol. 1 janv 2013;14(1):55-63.
